# Applications of Circulating Tumor DNA in Immune Checkpoint Inhibition: Emerging Roles and Future Perspectives

**DOI:** 10.3389/fonc.2022.836891

**Published:** 2022-03-14

**Authors:** Chang Lu, Yi-Chen Zhang, Zhi-Hong Chen, Qing Zhou, Yi-Long Wu

**Affiliations:** Guangdong Lung Cancer Institute, Guangdong Provincial People’s Hospital, Guangdong Academy of Medical Sciences, Guangzhou, China

**Keywords:** liquid biopsy, immune checkpoint inhibition, immunotherapy, lung cancer, noninvasive biomarker, circulating tumor DNA

## Abstract

Immune checkpoint inhibitors (ICIs), especially anti-programmed death 1 (PD-1)/programmed death ligand 1 (PD-L1) antibodies, have made dramatic progress in the treatment of lung cancer, especially for patients with cancers not driven by oncogenes. However, responses are limited to a subset of patients, and which subset of patients will optimally benefit from ICI remains unknown. With the advantage of being minimally invasive and dynamic, noninvasive biomarkers are promising candidates to predict response, monitor resistance, and track the evolution of lung cancer during ICI treatment. In this review, we focus on the application of circulating tumor DNA (ctDNA) in plasma in immunotherapy. We examine the potential of pre- and on-treatment features of ctDNA as biomarkers, and following multiparameter analysis, we determine the potential clinical value of integrating predictive liquid biomarkers of ICIs to optimize patient management. We further discuss the role of ctDNA in monitoring treatment resistance, as well as challenges in clinical translation.

## Introduction

Immune checkpoint inhibitors (ICI), especially anti-PD-1/PD-L1 antibodies, have been widely used as an effective therapy for many types of cancer, with impressive long-lasting responses in patients with non-small cell lung cancer (NSCLC) (1–3). However, only a minority of patients derive durable clinical benefit from ICIs (4). In addition, by increasing the activity of the host’s immune system to kill tumors, ICIs also cause toxicity known as immune-related adverse events (irAEs), which can result in the discontinuation of ICIs and, in some cases, these reactions can be life-threatening (5–7). Furthermore, the unique nature and timing of the responses to ICIs represent a challenging scenario for the currently available radiologic methods. Therefore, the prediction of response and toxicity to ICI before or early during the treatment course could help identify potential durable responders while sparing non-responders or those at risk of experiencing higher toxicity from ICIs.

The current gold standard of molecular profiling still relies on tissue samples. Although several tumor characteristics including tumor PD-L1 expression, tumor mutational burden (TMB), and microsatellite instability (MSI) status have been heavily studied, robust tumor-based predictive markers of treatment response remain elusive (4, 8–12). In addition, repeated tumor biopsies in the treatment course are usually not feasible and are limited by the quality of the specimen and intratumoral heterogeneity (ITH), presenting an unmet clinical need. This requires the identification of noninvasive biomarkers that can help to direct treatment decisions, gauge subsequent responses, and alert to the emergence of treatment resistance to PD-1/PD-L1 blockade-based ICIs.

The term “noninvasive biomarker” refers to a component from a variety of biospecimens, most notably available in the blood, as well as other clinical specimens such as urine, saliva, stool, and cerebrospinal fluid (13–17). In the search for putative noninvasive biomarkers, blood remains the most collected and studied sample. Peripheral blood can identify biomarkers including circulating immune cells, cytokines, soluble proteins [e.g., soluble cytotoxic T lymphocyte-associated protein 4 (CTLA-4) and soluble PD-1/PD-L1], circulating tumor cells, and tumor cell-derived material (DNA, RNA, and exosomes) (18–21). This review focuses on a widely studied application of circulating tumor DNA (ctDNA), together with multiparameter models integrating predictive liquid biomarkers of ICI treatment outcome. We summarize the implementation of ctDNA in the context of ICI treatment in patients with NSCLC. We further discuss the potential of ctDNA to predict outcomes and to dynamically monitor treatment response with a particular emphasis on challenges in clinical translation.

## Emerging Applications of ctDNA for Immune Checkpoint Inhibitors

Numerous candidate noninvasive biomarkers have emerged in the past few years. The leading example of a plasma predictive biomarker is ctDNA, which refers to tumor-derived DNA fragments in cancer patients. ctDNA harbors characteristic somatic genomic alterations, the patterns of nucleosome occupation that suggest the tissue of origin, and DNA methylation profiles (22–25). Therefore, ctDNA can be used to identify tumor-specific genetic alterations including somatic point mutations, loss of heterozygosity, structural variants, and epigenetic alterations, including DNA methylation differences, with future potential for predictive biomarker development. NSCLC is a cancer for which plasma ctDNA testing has the most compelling and comprehensive evidence.

### Using Pretreatment Features for Early Prediction of Response and Toxicity

Prediction of response and toxicity with ICI before treatment could spare nonresponders or those at risk of higher toxicity from unnecessary misery and cost. In this section, we discuss ctDNA metrics before treatment for risk stratification and identification of response and toxicity.

Several studies have established the predictive power of pretreatment plasma ctDNA level, and a high pretreatment ctDNA level was an independent predictor of unfavorable outcome across multiple types of cancer (26–28). Associations with baseline ctDNA levels were observed in a prospective phase II clinical trial (NCT02644369), including a cohort of patients with advanced NSCLC treated with pembrolizumab (29). Lower baseline ctDNA levels than the median were associated with better overall survival [hazard ratio adjusted for cohort (aHR) 0.49, 95% confidence interval (CI) 0.29–0.83] and progression-free survival (PFS; aHR 0.54, 95% CI 0.34–0.85). We envision that pretreatment characteristics could stratify higher risk cancers with higher ctDNA shed, which may identify patients requiring more aggressive treatment strategies, as can be seen in trials in which patients with high plasma ctDNA levels are directed toward more intensive therapy than their counterparts with low plasma ctDNA levels (**Table 1**). Similar predictive values have also been reported in blood-based assays to measure blood-based tumor mutational burden (bTMB) levels in large randomized clinical trials, POPLAR, OAK, and MYSTIC (30, 31). An improved benefit for ICI treatment was observed in NSCLC patients with higher bTMB cutoff values, with a bTMB >16 mut/Mb demonstrating a median overall survival of 13.5 months for atezolizumab vs. 6.8 months with docetaxel in the OAK study. Interestingly, the authors determined that the maximum somatic allele frequency (MSAF) of ctDNA, which reflects the ctDNA amount in the blood, could interfere with TMB results. Wang et al. (32) further adjusted bTMB for MSAF, and they demonstrated that MSAF of ctDNA could provide an additional predictive value for bTMB.

**Table 1:** Interventional plasma-adapted trials of immune checkpoint inhibitors in NSCLC.

Clinicaltrials.gov identifier	Sponsor	Phase	Subject	Experimental	Change threshold	Time point	Primary endpoint
NCT04166487	Dana-Farber Cancer Institute	2	Stage IV, untreated	Pembrolizumab for 2 cycles, following pembro (with plasma response) or pembro + chemo (without plasma response)	Patients with high shed [≥0.5% max AF] at C1D1: ≥50% reduction in plasma ctDNA max AF; patients with low shed [<0.5% max AF] at C1D1: persistent low shed	C2D1	6-month progression-free survival rate
NCT04093167	Canadian Cancer Trials Group	2	Stage IV	Pembrolizumab	NA	NA	Concordance rate between molecular response and radiologic response
NCT04367311	Nasser Hanna	2	Stage I (T ≥4 cm), IIA, IIB (and select IIIA); detectable ctDNA after surgery	Adjuvant atezolizumab + chemotherapy for 4 cycles following up to 13 cycles of atezolizumab	ctDNA clearance [CAPP-seq, using the Monte Carlo-based ctDNA detection index cutoff point of < 0.05]	Landmarks (after 4, 8, 12, 17 cycles)	Percentage of patients with undetectable ctDNA
NCT04642469	AstraZeneca	3	Stage II–III, MRD+ following curative intent therapy	Durvalumab (control: placebo)	Minimal residual disease (using personalized ctDNA assays)	During a 96-week surveillance period	Disease-free survival

Abbreviations: pembro, pembrolizumab; AF, allele frequency; C1D1: cycle1 day 1; NA: not available; ctDNA, circulating tumor DNA; CAPP-seq, cancer personalized profiling by deep sequencing; MRD, minimal residual disease.

Several studies have explored the associations between peripheral blood markers and the onset of irAEs in patients with advanced NSCLC receiving ICIs, with T-cell receptor diversity, CD8+ T-cell clonal expansion, peripheral immune cells, cytokines, preexisting antibodies (33–37), and the circulating microbiome (38–40), which all represent attractive biomarkers. Previous studies have demonstrated that TMB (41) and genes related to T-cell activation (42) could be potential biomarkers to predict irAEs. However, ctDNA, which allows analysis of genetic features, has not been explored as a predictive marker for irAE. Three reasons should be considered. First, these potential irAE predictors were conducted in a limited number of cases and need to be validated; second, these results were conducted with tissue samples instead of plasma samples; third, technical challenges remain on analyzing genomic traits using plasma-based approaches. Further studies are warranted to enable comprehensively predicting irAE.

### Long-Term Longitudinal Monitoring of Treatment Response and Resistance

In this section, we discuss the utility of early on-treatment (usually within 8 weeks after treatment initiation) and extended monitoring, as well as the potential of plasma ctDNA to be used as a possible adjunct to radiographic assessment, among studies that utilize noninvasive biomarkers for long-term longitudinal monitoring response to checkpoint blockade and prediction of the risk of eventual progression.

#### Early On-Treatment Kinetics

The early response pattern (usually within 8 weeks after treatment initiation) during ICI treatment was recently shown to identify patients with NSCLC responding to therapy, regardless of the stage of the disease. In patients with advanced NSCLC, several groups have recently demonstrated that an early reduction in ctDNA allele frequency, also known as molecular response, was independently associated with longer survival (PFS and overall survival) and a higher response rate [objective response rate (ORR): complete or partial response] in patients with NSCLC treated with durvalumab (43) and pembrolizumab ± chemotherapy (overall survival: aHR 0.36, 95% CI 0.18–0.7; PFS: aHR 0.33, 95% CI 0.19–0.58) (29, 44), as well as other ICIs (45, 46). In contrast, an early increase in ctDNA level was associated with increased tumor volume and prolonged duration of treatment in these studies, with landmark time points ranging from within the first 8 weeks to up to 12 weeks on-treatment.

A particular conundrum in clinical practice is the discrimination between patients with early radiological stable disease who would truly benefit from treatment from those who would not. Recent studies have reported that ctDNA responses within 8 weeks after starting treatment could help determine the likelihood of durable clinical benefit (defined as complete response, partial response, or stable disease for 6 months) from ICIs (43, 45, 47). In an advanced NSCLC study, ctDNA responses after a single cycle of ICI therapy distinguished most patients with long-term benefits. Interestingly, early ctDNA dynamics outperformed all individual pretreatment factors (P < 0.05, accuracy = 73%) in classification of durable benefit (48).

Intriguingly, in the aforementioned study, when ctDNA was reduced to undetectable (also known as ctDNA clearance), superior clinical outcomes were observed; all patients with ctDNA clearance during treatment experienced prolonged duration of objective response and were alive with a median of 25 months of follow-up (29).

Furthermore, recent data on resectable NSCLC support ctDNA as a response biomarker in neoadjuvant ICI treatment (49). In the CheckMate-816 study, a phase III study exploring the efficacy of neoadjuvant nivolumab in stage IB–IIIA NSCLC, ctDNA clearance on day 1 cycle 3 post-ICI treatment was associated with a pathological complete response (50). In addition, in patients with unresectable locoregionally advanced NSCLC, ctDNA analysis has also shown promise to indicate whether further treatment is needed and could identify responders. Moding et al. (51) applied CAPP-Seq ctDNA analysis and found that in patients with detectable ctDNA after chemoradiation therapy, those who received consolidation immune checkpoint inhibition had significantly better outcomes than those who did not, and patients with decreasing ctDNA early during treatment derived superior outcomes than those with increased ctDNA levels. Future interventional studies will be required to enable clinical decisions using ctDNA to guide ICI treatment after curative intent treatment, including surgery and chemoradiation therapy.

#### Dynamic Changes Throughout the Course of the Treatment

Consistent with early on-treatment ctDNA kinetics, distinct ctDNA dynamic profiles during surveillance were also correlated with benefit. Longitudinal monitoring of ctDNA throughout the course of treatment in a study of advanced NSCLC also demonstrated that an increase in ctDNA level from baseline was associated with disease progression and poor survival (29). Furthermore, in the aforementioned study, clearance of ctDNA could occur at any time point from cycle 3 to cycle 12, suggesting that extended surveillance could add to the clinical utility of ctDNA-based monitoring.

#### Combining Imaging Findings With ctDNA Monitoring in Response Assessment

The unique nature and timing of the responses to ICIs represent challenging scenarios for current radiologic methods. Plasma ctDNA levels have been correlated with tumor burden and thus may promptly and accurately assess clinical responses and disease progression in response to ICI treatment (47, 52). Recently, emerging evidence has supported longitudinal changes in ctDNA levels that are consistent with and precede changes observed on radiographic imaging of tumor size. Goldberg et al. (45) observed a strong agreement between ctDNA response and radiographic response in patients with advanced NSCLC receiving ICI treatment (Cohen’s kappa, 0.753), and this relative decrease in the fraction of mutant alleles from baseline translates to superior survival outcomes. They also found that among patients who achieved responses to ICIs, it takes a shorter time to assess initial response by ctDNA than by imaging (median time to initial response, 24.5 vs. 72.5 days).

ctDNA dynamics may also serve as a potential marker to identify pseudoprogression. Pseudoprogression represents another challenging scenario for current radiologic methods, as it remains difficult to recognize accurately or promptly, and patients may discontinue treatment that can eventually be effective and extend survival (53–55). One of the first studies to highlight the utility of ctDNA in the identification of pseudoprogression was reported by Lee et al. (56) following the analysis of a cohort of 125 patients with stage IV melanoma who received pembrolizumab or nivolumab either alone or in combination with ipilimumab. The ctDNA profile could discriminate pseudoprogression from true progression with a sensitivity of 90% and a specificity of 100%. The use of ctDNA to identify this unique response pattern has also been explored for NSCLC. In a previously published case report, Guibert et al. (57) found a rapid and dramatic decrease in ctDNA levels in two patients exhibiting pseudoprogression, whereas there was an increase in a true-progressive patient.

#### Early Warning of Disease Progression

Despite the initial response, and even long-lasting response to ICI treatment, a substantial fraction of patients eventually progress. An emerging field using a noninvasive biomarker is in its longitudinal deployment as an early warning of acquired resistance after immune checkpoint blockade, which can direct early intervention in those at highest risk for eventual progression. The kinetic patterns of ctDNA could provide added clinical utility beyond imaging in two aspects. First, in long-term responders, ctDNA analysis could be used to predict the risk of eventual disease progression on ICI treatment. In a recent study on a cohort of patients with NSCLC achieving long-term benefit from PD-1/PD-L1 blockade, patients with positive detection of ctDNA at late surveillance time points were more likely to develop disease progression. Almost all patients with undetectable ctDNA at the surveillance blood draw remained disease-free, while all of the patients with detectable ctDNA eventually progressed (58). Second, in patients without radiological progression, ctDNA has the potential to inform acquired resistance before imaging findings. In the TRACERx study, Abbosh et al. (59) followed NSCLC patients after surgery and detected ctDNA with a median interval of 70 days prior to the identification of clinical relapse by computed tomography imaging. Furthermore, in a phase II trial evaluating the efficacy of ICIs in solid tumors of patients presenting radiological progression at a certain time point, those with decreased ctDNA levels showed longer survival than those with increased ctDNA. In addition, the combination of ctDNA dynamics and Response Evaluation Criteria in Solid Tumors (RECIST) improved the accuracy of Cox models for overall survival over that of RECIST alone (C statistic 0.62 vs. 0.67, likelihood ratio test P = 0.02) (38), suggesting that ctDNA dynamics could serve as a reliable molecular biomarker to help determine whether therapeutic interventions should continue after radiological progression to improve patient outcomes. Longitudinal blood samples may also anticipate in resistance mechanism exploring and subsequent functional testing, as suggested by paired models generated from SCLC patients representing the disease before and after the development of resistance to therapy (60–62).

Collectively, these promising results indicate that longitudinal tracking with blood samples has great potential in monitoring treatment response and resistance more nimbly than imaging in patients treated with ICIs, thus allowing more timely systemic treatment changes for individual patients.

### Early Development in Integrating ctDNA With Other Parameters for Predicting Response to Immune Checkpoint Inhibitor Treatment

In the CheckMate-816 study, more than 50% of patients with ctDNA clearance did not reach a pathological complete response after immunotherapy (50), suggesting the limit of prediction based on a single tumor characteristic. Nabet et al. (48) and Zhang et al. (52) have recently demonstrated that multivariate models based on pretreatment ctDNA and peripheral immune features, together with early on-treatment ctDNA dynamics, robustly predict durable responders with higher accuracy than any individual feature alone. This finding represents an important concept that incorporating pretreatment features in addition to time-dependent on-treatment changes can support biological plausibility, which enables a personalized approach to longitudinally refine risk stratification. Similarly, a recent report of a phase II trial evaluated immune checkpoint blockade, in which the association with PFS, overall survival, clinical response, and clinical benefit became stronger when integrating both baseline ctDNA concentration and ctDNA kinetics during treatment (29). Moreover, incorporating nonliquid components like tumor features could provide additional predictive value. NSCLC has been shown to harbor significant ITH, especially after the onset of resistance to therapy (63). Recently, Fang et al. (64) reported ITH as a predictive biomarker in anti-PD-1/PD-L1 therapy for NSCLC. We believe that integrating dynamic multiparametric biomarkers provides a significant advantage over traditional static single-metric modeling in predicting the response orchestrated by both the tumor and the immune milieu and represents promising developments in the hunt for noninvasive biomarkers in response to ICI immunotherapies in NSCLC patients. Further refinement of the components of the model remains an open challenge, requiring prior knowledge derived from previously published datasets and cross-study validation.

### Tracking the Co-Evolution of Cancer and Antitumor Immunity Following Exposure to Immune Checkpoint Inhibitor Perturbations

The co-evolution of tumor and the immune microenvironment during ICI treatment is receiving growing interest. ctDNA-based monitoring has been reported to help provide a longitudinal evaluation of tumor genetic clonal characteristics (59) and inform changes of neoantigens and neoantigen-producing mutations throughout the course of ICI treatment (65, 66); thus, this can be leveraged to track the co-evolution of tumor and immune microenvironment. First, the neoantigens of tumors may reshape the host immune repertoire, which reflects the antitumor immune response (67). Next, through either the elimination of tumor subclones or the expansion of resistant clones, the selective pressures created by ICIs may have an impact on the clonotypic expansion of neoantigen-specific T cells (47, 68–70). Moreover, tumor genetic evolution may be in turn influenced by antitumor immunity by imposing a selection pressure through affecting neoantigens and antigen presentation (65). With a deeper understanding of the co-evolution, ctDNA-based monitoring can help provide insight into the prediction of response and reveal the mechanism of resistance to ICI.

## On the Horizon, Challenges to Be Overcome

Given the variety of noninvasive biomarkers and their diverse natures and predictive values, translating their use into clinical reality remains an unmet need. Although ctDNA levels and dynamics were believed to be the most predictive of immunotherapy response and are being intensively studied, a harmonized threshold for risk stratification at baseline is lacking, let alone diverse detecting approaches with different consistent depths of coverage, fragmentation sizes, and limits of detection (71). Furthermore, to define molecular responses, variable ctDNA change thresholds [variable levels of reduction (29, 52) or reductions >50% (48) and time points (ranging from within the first 8 weeks (47, 48) to up to 12 weeks on-treatment (29)] have been used in different studies, while a consensus definition remains elusive (72), leaving questions about the most effective “sweet spot”. In monitoring acquired resistance, numerous open challenges also remain, including determining which variants should be tracked and which filtering out as variants arising from clonal hematopoiesis. A variety of other components of the immune response are under active investigation. However, technical obstacles still remain.

Although a dynamic multiparameter model might outperform the predictive ability of any single feature, incorporating different components relies on the harmonization of diverse assays and multiple selective biomarkers will be crucial. Incorporating non-liquid components like radiomic imaging analysis and tumor features (73), coupled with multidimensional approaches involving blood-based proteomic testing (74) and mechanistic learning (75, 76), is also required. Additionally, consistent cross-study validation and standardization of each model are required before any implementation in routine clinical use to improve personalized medicine approaches, which could be addressed through ongoing prospective collaborative molecular response-adaptive clinical trials (**Table 1**).

## Conclusions

**Figure 1:**
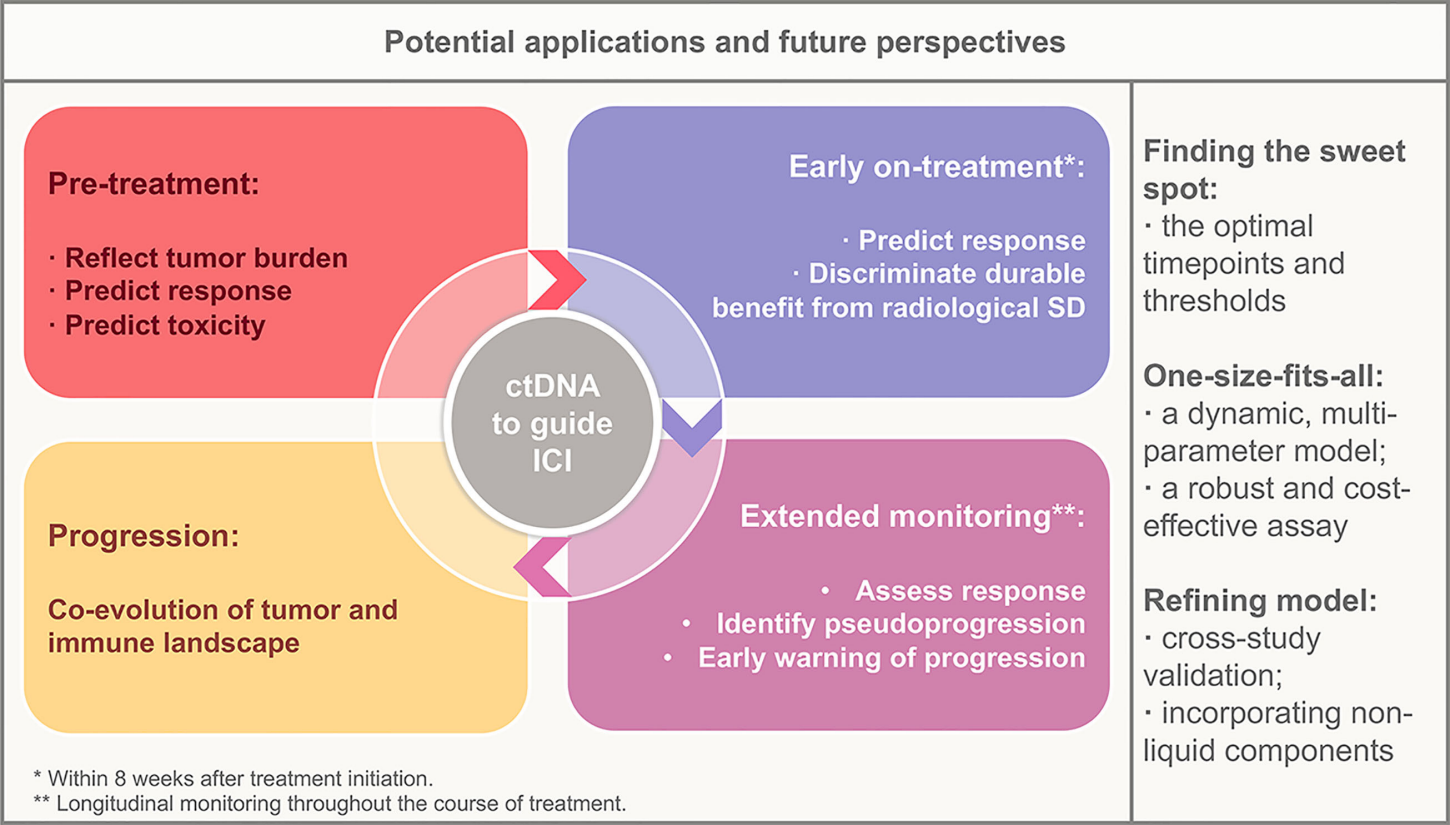
Potential applications of circulating tumor DNA in the context of immunotherapy and future perspectives. ctDNA, circulating tumor DNA; SD, stable disease.

ctDNA heralds a revolution in the broader application of biomarker-directed ICI treatment. Early identification of therapeutic benefits and toxicity, as well as longitudinal noninvasive monitoring of therapeutic response and resistance in patients with ICI treatment, is an emerging approach to maximize the personalized benefit of ICIs. Although the current application of ctDNA in clinical practice remains limited, applications toward early identification of responders and response assessment are in the near future, and applications toward resistance monitoring and evolution tracking will be the next frontier (**Figure 1**).

Given the emerging developments in ctDNA, the notion that liquid biopsies could support the management of patients receiving immunotherapy is exciting, especially when pseudoprogression or higher risk of disease progression is identified, and a timely treatment decision can be made accordingly. Innovative clinical trials incorporating dynamic noninvasive biomarkers into ICI treatment monitoring will enable personalized ICI treatment care for patients with NSCLC.

## Author Contributions

Conception and design: All authors. Collection and assembly of data: All authors. Data analysis and interpretation: CL, Y-CZ, QZ, and Y-LW. Article writing. CL, Y-CZ. Article revision: Z-HC, QZ, Y-LW. Final approval of article: All authors. Accountable for all aspects of the work: All authors.

## Funding

This work was supported by the research fund from Guangzhou Science and Technology Bureau (grant no. 202102021154 to Y-CZ), the National Natural Science Foundation of China (grant no. 82102808 to Y-CZ), and the Key Lab System Project of Guangdong Science and Technology Department—Guangdong Provincial Key Lab of Translational Medicine in Lung Cancer (Grant No. 2017B030314120 to Y-LW).

## Conflict of Interest

QZ reports honoraria from AstraZeneca, Boehringer Ingelheim, BMS, Eli Lilly, MSD, Pfizer, Roche, and Sanofi, outside the submitted work. Y-LW reports advisory services for AstraZeneca, Boehringer Ingelheim, Novartis, Takeda; personal fees from AstraZeneca, Beigene, Boehringer Ingelheim, BMS, Eli Lilly, MSD, Pfizer, Roche, Sanofi; and grants from AstraZeneca, Boehringer Ingelheim, BMS, Hengrui, and Roche, outside the submitted work.

The remaining authors declare that the research was conducted in the absence of any commercial or financial relationships that could be construed as a potential conflict of interest.

## Publisher’s Note

All claims expressed in this article are solely those of the authors and do not necessarily represent those of their affiliated organizations, or those of the publisher, the editors and the reviewers. Any product that may be evaluated in this article, or claim that may be made by its manufacturer, is not guaranteed or endorsed by the publisher.
